# A brief review of peer review in DMM in 2019

**DOI:** 10.1242/dmm.044172

**Published:** 2020-01-24

**Authors:** Rachel Hackett

During 2019, Disease Models & Mechanisms (DMM) partnered with Publons to trial their Reviewer Recognition tool, giving reviewers formal recognition of their peer review contributions. Reviewers can now choose to add their DMM review to their Publons profile when completing the reviewer form (via an automated process). The profile can then be used in job, visa and grant applications, complete with verified review activities. Since the tool's launch, more than 40% of DMM's reviewers have taken up this option (data from Publons) and we hope that more will do so.

DMM is excited to be an affiliate journal for Review Commons, a new platform for high-quality journal-independent peer review in the life sciences. Reviewers will be asked to focus on the science rather than fit for a journal. DMM Editors have agreed to consider articles and their transferred reviews without soliciting additional reviews (although additional expert advice might be necessary on occasion). We look forward to receiving submissions from the Review Commons platform.

Following a successful trial, DMM now operates a system of cross-referee commenting, to help resolve differences between referees, identify unnecessary or unreasonable requests, or – conversely – highlight valid concerns raised by one referee but overlooked by others. Happily, average speeds to decision and acceptance have not been affected by this change.

The Company of Biologists and DMM strive to support the academic community by engaging a broad and diverse array of authors, reviewers, Editors, editorial staff, editorial board members and readers in their activities. DMM encourages authors and Editors to consider diversity in career stage, geographical location, gender and ethnicity when suggesting and selecting appropriate reviewers for a manuscript. This was prompted, in part, by a gender analysis conducted across The Company of Biologists' journals (see Box 1). More recently, the issue of ghost writing of peer reviews by junior researchers was highlighted. For early-career scientists to be involved in the peer review process, DMM requires that there must be a genuine mentoring process and that the senior invited reviewer should always take final responsibility for the report delivered to DMM. The name of the co-reviewer must be reported to the Editor; a field is provided in the report form for this purpose. The names of these co-reviewers are also included in our annual published list of reviewers (see Supplementary Information). We thank every one of them for their expertise and time, as well as our authors, readers and Editors for their support.

**Box 1. Gender analysis across The Company of Biologists' journals.**

This analysis was done primarily by Sam Holden, a PhD student at the University of Norwich, who in 2018 spent three months as a Professional Internships for PhD Students (PIPS) intern with us as part of his PhD program. Sam was helped in his analysis by the Royal Society of Chemistry (RSC), who developed the algorithm used to assign gender to names (and have recently published detailed statistics on gender bias: https://www.rsc.org/globalassets/04-campaigning-outreach/campaigning/gender-bias/gender-bias-report-final.pdf). Many thanks to Sam for his work on this project, and to our colleagues at the RSC for their support.

Methodology

For each of our five journals (Development, Journal of Cell Science, Journal of Experimental Biology, Disease Models & Mechanisms and Biology Open), we downloaded data on all research papers submitted between October 2006 and May 2018, and extracted the following information:

- Outcome of submission (editorially rejected, rejected post-review or accepted)
- Name of first author
- Name of corresponding author (note that this may be the same as the first author)
- Names of individuals suggested by authors as potential reviewers
- Names of individuals invited to review the paper
- Names of reviewers who completed a report on the paper

We ran the lists of names through an algorithm that assigns gender to names, along with a confidence value in the assignment. We assigned a gender to names where the confidence value was greater than 90%, allowing us to assign gender to ∼75% of authors and 85% of reviewers. It should be noted that the algorithm was developed using a dataset of mainly Western names, and the majority of names with ‘unassigned’ gender are Asian. Thus, the results outlined below do not necessarily reflect patterns that might apply to non-Western authors and reviewers.

To allow more rigorous statistical analysis, data were pooled across all the journals and the whole >10-year time span, although we have also looked at trends over time and between journals.

In addition to calculating basic statistics on the gender balance of our author and reviewer pool, we also analysed the success rate of submissions based on author and reviewer gender.

Key results (combined data for all five Company journals)

- Almost exactly 50% of first authors (typically the junior researchers who contributed most to the research) are female – implying minimal gender disparity at the level of the PhD students and postdocs in our community of authors. However, among corresponding authors (typically principal investigators/lab heads) only 30.3% were female.
- The gender of the first author had no influence on the success rate of the submission. However, papers from female corresponding authors showed a slight, but statistically significant (*P*<0.05), reduction in acceptance rate – only 28.5% of corresponding authors on accepted papers were female.
- Disparity is seen at both initial editorial assessment and at peer review: papers with female corresponding authors are less likely to be sent out for peer review than those with male corresponding authors (67.3% versus 71.0%) and, once sent out for peer review, are less likely to be accepted for publication (52.9% versus 56.2%).
- There is a greater gender imbalance in our pool of reviewers than in our pool of corresponding authors: 26.1% of people invited to review a paper are female and 25.8% of completed reviews are by women (the similar numbers suggesting that both genders are equally likely to accept an invitation to review). These figures have improved over the 10-year time window: in 2007, only 23% of reviewers were female; this reached 29% by 2017 (though this is still below the 30% proportion of female corresponding authors).
- Authors are more likely to suggest reviewers of the same gender as themselves. However, we have not found evidence that female-authored papers are at a disadvantage if reviewed by men (although the data on correlations between author and reviewer gender are hard to interpret).

This box has also been reproduced in other Company journals. See also an Editorial (Briscoe and Brown, 2020) from the Development team that explores the subject in greater depth.

## Supplementary Material

Supplementary information
