# Supplementary material for: A brief review of peer review in DMM in 2019
Source: Dis Model Mech. 2020 Jan 24;13(1):dmm044172. doi: 10.1242/dmm.044172 (PMC6994959; doi:10.1242/dmm.044172)
Supplement: Supplementary information [file dmm-13-044172-s1.pdf]

Reviewers for Disease Models & Mechanisms 2019

Susan Abmayr, Stowers Institute for Medical Research, USA

Robert Abramovitch, Michigan State University, USA

Usha Acharya, University of Massachusetts Medical School, USA

James Amatruda, Children's Hospital Los Angeles, USA

Bogi Andersen, University of California, Urvine, USA

Suhail Andrabi, Lerner Research Institute, USA

Guiseppina Andreotti, Instituto di Chimica Biomolecolare-CNR, Italy

Jonathan Andrews, Baylor College of Medicine, USA

Lynda Aoudjehane, Institute of Cardiometabolism and Nutrition (ICAN), France

Dean Appling, University of Texas Austin, USA

Virginia Arechavala-Gomeza, Biocruces Bizkaia Health Research Institute, Spain

David Arnold, Ohio State University, USA

Atsushi Asakura, Stem Cell Institute, University of Minnesota, USA

Miriam Baes, KU Leuven, Belgium

Herwig Baier, Max Planck Institute of Neurobiology, Germany

Jeroen Bakkers, Hubrecht institute, The Netherlands

Volodymyr Balatskyi, Nencki Institute of Experimental Biology, Poland

Oliver Bandmann, University of Sheffield, UK

Thomas Baranski, Washington University, USA

Isabelle Baro, University of Nantes, France

Jugajyoti Baruah, Harvard Medical School, USA

Gill Bates, UCL Institute of Neurology, UK

Aaron Beedle, SUNY Binghamton University, USA

Hugo Bellen, HHMI - Baylor College of Medicine, USA

Melina Bellin, Leiden University Medical Center, The Netherlands

Susan Bellis, University of Alabama-Birmingham, USA

Dylan Bergen, University of Bristol, UK

Joachim Berger, ARMI, Monash University, Australia

Jason Berman, Dalhousie University, Canada

Sanford Bernstein, San Diego State University, USA

Julien Bertrand, University of Geneva, Switzerland

Roberta Besio, University of Pavia, Italy

Colin Bingle, The University of Sheffield Medical School, UK

Benoit Biteau, University of Rochester Medical Center, USA

Karen Blyth, The Beatson Institute for Cancer Research, UK

Carla Boccaccio, Laboratory of Cancer Stem Cell Research, Istituto di Candiolo, Italy

Cornelius Boerkoel, University of British Columbia, Canada

Johann Bohm, Institut de Genetique et de Biologie Moleculaire et Cellulaire, France

Dirk Bohmann, University of Rochester Medical Center, USA

Cesario Borlongan, University of South Florida, USA

Alexander Borowsky, UC Davis Cancer Center, USA

Luke Boulter, University of Edinburgh, UK

Melissa Bowerman, University of Oxford, UK

Teresa Bowman, Albert Einstein College of Medicine, USA

Susan Brain, BHF Cardiovascular Centre of Excellence, King's College, London, UK

Andrea Brancaccio, ICRM, CNR Università Cattolica del Sacro Cuore, Italy

David Brenner, University of California San Diego, USA

Volker Briken, University of Maryland, USA

Elizabeth Brooks, Duke University Medical Center, USA

Marco Brotto, University of Texas, USA

Fiona Brown, Monash University, Australia

Leslie Bruggeman, Case Western Reserve University, USA

Liam Brunham, University of British Columbia, Canada

Valerie Brunton, University of Edinburgh, UK

Andrew Bryant, University of Florida Health, USA

Vladimir Buchman, University of Cardiff, UK

Robert Burgess, The Jackson Laboratory, USA

Shawn Burgess, NHGRI/NIH, USA

Brant Burkhardt, University of South Florida, USA

Dean Burkin, Univesrity of Nevada, Reno, USA

Tom Burne, Queensland Brain Institute, Australia

Claudio Cabello-Verrugio, Universidad Andres Bello, Chile

Kim Caldwell, The University of Alabama, USA

Tito Calí, University of Padova, Italy

Christopher Cambier, Stanford University, USA

Alberto Caminero, Farncombe Family Digestive Disease Research Institute, Canada

Ornella Cappellari, Royal Veterinary College, UK

Carlos Carmona-Fontaine, New York University, USA

Thomas Carroll, University of Texas Southwestern Medical Center, USA

Tamara Caspary, Emory University, USA

Craig Ceol, University of Massachusetts Medical School, USA

Jeffrey Chamberlain, University of Washington, USA

Connie Chamberlain, University of Wisconsin, Madison, USA

Danny Chan, The University of Hong Kong, Hong Kong

Wood Yee Chan, The Chinese University of Hong Kong, Hong Kong

Jichao Chen, MD Anderson Cancer Center Houston, USA

Ju Chen, University of California, San Diego, USA

Kong Chen, University of Pittsburgh, USA

Lisheng Chen, University of Michigan Kellogg Eye Center, USA

Sheng Chen, Zhejiang University, China

Weiqin Chen, Augusta University, USA

Wenbiao Chen, Vanderbilt University, USA

Xing-Zhen Chen, University of Alberta, Canada

Yu-Shan Cheng, NIH, USA

Jen-Tsan Chi, Duke University School of Medicine, USA

Alexandre Chlenski, University of Chicago, USA

Ginam Cho, Harvard Medical School, USA

Clement Chow, University of Utah School of Medicine, USA

Yuen-Li Chung, The Institute of Cancer Research, UK

Michael Clarke, Stanford University, USA

Hans Clevers, Hubrecht Institute, The Netherlands

David Clouthier, University of Colorado, Denver, USA

Holly Colognato, SUNY Stony Brook University, USA

Victoria Connaughton, American University, USA

Thomas Cooper, Baylor College of Medicine, USA

Callie Corsa, University of Michigan, USA

Belinda Cowling, Dynacure, France

Roger Cox, Medical Research Council Harwell Institute, UK

Timothy Cox, University of Missouri, USA

Timothy Cox, Sidney Sussex College, UK

Peter Crouch, University of Melbourne, Australia

Gage Crump, University of Southern California, USA

Salvatore Cuzzocrea, University of Messina, Italy

Christian Dahmann, Dresden University of Technology, Germany

Rodney Dale, Loyola University Chicago, USA

Revati Darp, University of Massachusetts, USA

Mark Davenport, King's College Hospital, UK

Neil Dawson, University of Lancaster, UK

Francesca De Bacco, Istituto di Candiolo, Italy

Pietro De Camilli, Yale University/Howard Hughes Medical Institute, USA

Jose de Celis, Universidad Autónoma de Madrid, Spain

Susana De la Luna, Centre for Genomic Regulation (CRG), Spain

Anna-Maria De Luca, University of Bari Aldo Moro, Italy

Francesca De Santa, National Research Council, Italy

April DeLaurier, USC Aiken, USA

Christos Delidakis, Forth Institute of Molecular Biology and Biotechnology, Greece

Thierry Delzescaux, Commissariat à l'Energie Atomique, France

Alexis Demonbreun, Northwestern Medicine, USA

Nicolas Denans, Stowers Institute, UK

Qing Deng, Purdue University, USA

Donna Denton, University of South Australia, Australia

Emily Derbyshire, Duke University School of Medicine, USA

Evandro De-Souza, Universidade Federal do Rio de Janeiro, Brazil

Stephen Devoto, Wesleyan University, USA

Simone Di Giovanni, Imperial College London, UK

Elia Di Schiavi, Institute of Biosciences and BioResources, Napoli, Italy

Albena Dinkova-Kostova, University of Dundee, UK

Maziar Divangahi, McGill University, Canada

James Dowling, Hospital for Sick Children, Canada

Zdenek Drahota, Institute of Physiology (IPHYS), Czech Republic

Elodie Drapeau, Icahn School of Medicine at Mount Sinai, USA

Pierre Drapeau, Université de Montréal, Canada

Shaojun Du, University of Maryland Biotechnology Institute, USA

Dongsheng Duan, University of Missouri, USA

Aditi Dubey, University of Maryland, USA

James Duce, University of Leeds, UK

Michael Duchen, University College London, UK

Debdeep Dutta, Baylor College of Medicine, USA

Tatiana Egorova, Institute of Gene Biology, Russian Academy of Sciences, Russia

Judith Eisen, University of Oregon, USA

Karin Eisinger, University of Pennsylvania, USA

Eran Elinav, Weizmann Institute, Israel

Stone Elworthy, University of Sheffield, UK

Janice Endsley, University of Texas Medical Branch, USA

Charis Eng, Cleveland Clinic, Lerner Research Institute, USA

Christoph Englert, Leibniz Institute on Aging (FLI), Germany

Robert Erickson, University of Arizona, USA

Todd Evans, Albert Einstein College of Medicine, USA

Kimberley Evason, Huntsman Cancer Institute, USA

Sarah Ewald, University of Virginia, USA

Walid Fakhouri, University of Texas Health Science Center at Houston, USA

Steven Farber, Carnegie Institution, USA

Hesso Farhan, University of Oslo, Norway

Eva Faurobert, Université Grenoble Alpes, France

Laura Feltri, University at Buffalo, Jacobs School of Medicine and Biomedical Sciences, USA

Hui Feng, Boston University, USA

Pedro Fernandez-Fúnez, University of Minnesota, USA

Javier Fernández-Ruiz, Complutense University, Spain

Beatrice Filippi, University of Leeds, UK

Richard Finnell, Baylor College of Medicine, USA

Anthony Firulli, Indiana University School of Medicine, USA

Jason Fish, University of Toronto, Canada

Matthew Fisher, Cold Spring Harbor Laboratory, USA

Mark Fishman, Harvard Stem Cell Institute, USA

Lisa Foa, University of Tasmania, Australia

Flavia Fontanesi, University of Miami, USA

Francois Foulquier, University of Lille, France

Nikolaos Frangogiannis, Albert Einstein College of Medicine, USA

Manfred Frasch, University of Erlangen Nuremberg, Germany

J. Kimble Frazer, University of Oklahoma Health Sciences Center, USA

David Fredricks, Fred Hutchinson Cancer Research Center, USA

Hudson Freeze, Sanford Burnham Prebys, Medical Discovery Institute, USA

Deborah French, University of Pennsylvania, USA

Sylvie Friant, University of Strasbourg, France

Máximo Galindo, Centro de Investigación Príncipe Felipe, Spain

Michael Galko, University of Texas MD Anderson Cancer Center, USA

Guangping Gao, University of Massachusetts Medical School, USA

Benjamin Gastfriend, University of Wisconsin, USA

Angela Gelli, University of California, Davis, USA

Martin Gering, Nottingham University, UK

Flavia Giamogante, University of Padova, Italy

Angela Giangrande, IGBMC, France

Claire Gibson, University of Nottingham, UK

Jonathan Glass, Emory University, USA

Joshua Goldberg, The Hebrew University of Jerusalem, Israel

Jeffrey Golden, Harvard University, USA

Heather Gordish-Dressman, Children's National Medical Center, USA

June Goto, Cincinnati Children's Hospital Medical Center, USA

Marie-José Goumans, Leiden University Medical Center, The Netherlands

Ryan Gray, University of Texas at Austin Dell Medical School, USA

Alex Gregorieff, McGill University, Canada

Miranda Grounds, The University of Western Australia, Australia

Majid Hafezparast, University of Sussex, UK

Chris Hall, University of Auckland, New Zealand

Robert Hammer, UT Southwestern, USA

Matthias Hammerschmidt, University of Cologne, Germany

Chrissy Hammond, University of Bristol, UK

Renzhi Han, The Ohio State University Wexner Medical Center, USA

Jonathan Hardy, Michigan State University, USA

Scott Harper, Nationwide Children's Hospital, USA

Peter Harris, Mayo Clinic, USA

Nicholas Hastie, Institute of Genetics and Molecular Medicine, University of Edinburgh, UK

Paul Hasty, The University of Texas Health Science Center at San Antonio, USA

Fenglei He, Tulane University, USA

Denis Headon, Roslin Institute, UK

Joan Heath, Walter and Eliza Hall Institute of Medical Research, Australia

Sarah Hescham, Maastricht University Medical Center, The Netherlands

Emma Heslop, John Walton Muscular Dystrophy Research Centre, UK

Eric Hewitt, University of Leeds, UK

Alicia Hidalgo, The University of Birmingham, USA

Susumu Hirabayashi, MRC Clinical Sciences Centre, Imperial College London, UK

Sevan Hopyan, The Hospital for Sick Children, Canada

Jason Horton, Upstate Medical University, USA

Huaiyu Hu, Upstate Medical University, USA

Ngan Huang, Stanford Medicine, USA

Neil Hukriede, University of Pittsburgh, USA

Dan Hultmark, Umea University, Sweden

Adam Hurlstone, University of Manchester, UK

Vera Hutchison, Baylor College of Medicine, USA

Tatsushi Igaki, Kyoto University, Japan

Marjan Iravani, University College London, UK

Clare Isacke, Breakthrough Breast Cancer Research Centre, UK

Junichi Iwata, The University of Texas Health Science Center at Houston, USA

Hamed Jafar-Nejad, Baylor College of Medicine, USA

Suh Young Jeong, Oregon Health and Science University, USA

Rulang Jiang, Cincinnati Children's Hospital, USA

Marvadita Jimenez Palomares, University of Michigan, USA

Suk-Won Jin, Yale University, USA

Jamie Johnston, University of Leeds, UK

Cameron Johnstone, Olivia Newton-John Cancer Research Institute, Australia

Tom Jongens, University of Pennsylvania School of Medicine, USA

Andrew Judge, University of Florida, USA

Marie Pierre Junier, Institute de Biologie Paris Seine, France

Monica Justice, Hospital for Sick Children, Canada

Takashi Kadowaki, The University of Tokyo, Japan

Philipp Kahle, University of Tübingen, Germany

Sharanya Kalasekar, University of Utah, USA

Junsu Kang, University of Wisconsin Madison, USA

Levente Kapás, Washington State University, USA

Ghaidaa Kashgari, University College Irvine, USA

Hidetaka Katow, NYU Langone Health, USA

Charles Kaufman, Washington University School of Medicine, USA

Deepak Kaushal, Texas Biomedical Research Institute, USA

Cristina Keightley, La Trobe University, Australia

Dan Kelpsch, Carnegie Institute for Science, USA

Robert A. Kesterson, University of Alabama at Birmingham, USA

Gausal Khan, Defence Institute of Physiology and Allied Sciences, India

Jean Kim, Baylor College of Medicine, USA

Min Sun Kim, Wonkwang University, Republic of Korea

Sung Eun Kim, The University of Texas at Austin, USA

Kerri Kinghorn, University College London, UK

Kassandra Kisler, University of Southern California, USA

Toshihiro Kitamoto, University of Iowa, USA

Yuliya Klymenko, Indiana University School of Medicine, USA

Joshua Knowles, Stanford University, USA

Kenji Kohno, Nara Institute of Science and Technology, Japan

Masaaki Koike, NAIST, Japan

David Kokel, University of California, San Francisco, USA

Yoshihiro Komatsu, The University of Texas Health Science Center at Houston, USA

Takefumi Kondo, Kyoto University, Japan

Stephen Konieczny, Purdue University, USA

Rashmi Kothary, Ottawa Hospital Research Institute, Canada

Boris Kramer, Maastricht University Medical Center, The Netherlands

Takayuki Kuraishi, Kanazawa University, Japan

Hiroshi Kurosaka, Osaka University Graduate School of Dentistry, Japan

Deborah Kurrasch, University of Calgary, Canada

Kristen Kwan, University of Utah, USA

Young Kwon, University of Washington, USA

Julia Ladewig, Central Insitute of Mental Health, Germany

Angela Laird, Macquarie University, Australia

Christina Lam, Seattle Children's Hospital, USA

Jennifer Lamberts, Ferris State University, USA

David Langenau, Massachusetts General Hospital, USA

Glenda Lassi, Society for Research on Nicotine and Tobacco, UK

Michael Lawlor, Medical College of Wisconsin, USA

Wei-Dong Le, Institute of Health Science, China

Fiona Le Beau, University of Newcastle-upon-Tyne, UK

Adrian V. Lee, University of Pittsburgh, USA

Disease Models & Mechanisms • Supplementary information

Jiae Lee, University of Washington, USA

Lance Lee, University of South Dakota, USA

Adele Lehane, Australian National University, Australia

Gregory Lesinski, Emory University, USA

Min Li, The University of Oklahoma Health Sciences Center, USA

Ye Li, The University of Chicago, USA

Yuqing Li, University of Florida, USA

Ellen Lien, University of Southern California, USA

Soren Lienkamp, University of Freiburg, Germany

Pei Hui Lin, Ohio State University, USA

Ethan Lippmann, Vanderbilt University, USA

Chuming Liu, Lucille Parker Markey Cancer Center, University of Kentucky, USA

Rick Livesey, University College London, UK

Hanns Lochmuller, CHEO Research Institute, Canada

Michael Lorenz, University of Texas Health Science Center, USA

Martin Lowe, University of Manchester, UK

Gigi Lozano, MD Anderson Cancer Center, USA

Pia Lundegaard, University of Copenhagen, Denmark

Yonglun Luo, Aarhus University, Denmark

Cathleen Lutz, The Jackson Laboratory, USA

Anisha Lynch-Godrei, University of Ottawa, USA

Long Ma, Central South University, China

Filippo Macchi, NYU Abu Dhabi, Abu Dhabi

Ormond MacDougald, University of Michigan, USA

Cressida Madigan, University of California, San Diego, USA

Pratyusha Mandal, Emory University, USA

Troy Markel, Indiana University School of Medicine, USA

Martin Marsala, University of California, San Diego, USA

Colin Martin, University of Alabama, USA

James Martin, Baylor College of Medicine, USA

Paul Martin, University of Bristol, UK

Alfonso Martín-Peña, University of Florida, USA

Emilia Martins, Arizona State University, USA

Andrea Martinuzzi, Istituto di Ricovero e Cura a Carattere Scientifico, Eugenio Medea Associazione, Italy

John Mason, University of Edinburgh, UK

Robert Maue, Dartmouth Geisel School of Medicine, USA

Lisa Maves, Seattle Children's Research Institute, USA

James McAllister, Washington University, USA

Jessica McCann, Duke University School of Medicine, USA

Kevin McCarthy, LSU Health Sciences Center, USA

Joseph McCarty, University of Texas MD Anderson, USA

Tara McCray, University of Illinois, USA

Steven McElroy, University of Iowa, USA

Jacqui McGovern, Queensland University of Technology, Australia

Kelly M. McNagny, University of British Columbia, Canada

Ashish Mehta, Victor Chang Cardiac Research Institute, Australia

Chris Mendias, Hospital for Special Surgery Research, USA

Marco Milan, IRB Barcelona, Spain

Rachel Miller, McGovern Medical School, USA

Yuji Mishina, University of Michigan, USA

Biswapriya Misra, Wake Forest School of Medicine, USA

Thimios Mitsiadis, Universität Zürich, Switzerland

Mayssa Mokalled, Washington University School of Medicine, USA

Jessica Momb, University of Texas Austin, USA

Satdarshan Monga, Institute of Pittsburgh, USA

Axel Montangne, Keck School of Medicine USC, USA

Sally Moody, George Washington University, USA

Lieve Moons, KU Leuven, Belgium

Mariya Moosajee, University College London, UK

Marie Morimoto, NIH, USA

Mitsuru Morimoto, RIKEN Center for Biosystems Dynamic Research, Japan

Simon Morley, University of Sussex, UK

Nuria Morral, Indiana University School of Medicine, USA

Jennifer Morton, The Beatson Institute, UK

Jenny Morton, University of Cambridge, UK

Christian Mosimann, University of Colorado School of Medicine, USA

Serge Mostowy, London School of Hygiene and Tropical Medicine, UK

Victoriano Mulero, Universidad de Murcia, Spain

Andrea Munsterberg, University of East Anglia, UK

Rohini Muthuswami, Jawaharlal Nehru University, India

Nael Nadif, Radboud UMC, The Netherlands

Lazlo Nagy, University of Debrecen, Hungary

Masanori Nakayama, Max Planck Institute for Heart and Lung Research, Germany

Mohandas Narla, New York Blood Centre, USA

Soumya Negi, University of Illinois, USA

Brent Neumann, Monash University, Australia

Alec Nickolls, National Institutes of Health, USA

Vincenzo Nigro, Telethon Institute of Genetics and Medicine (TIGEM), Italy

Natalia Ninkina, University of Cardiff, UK

Nikolay Ninov, Center for Regenerative Therapies, Dresden, Germany

Larisa Nonn, University of Illinois at Chicago, USA

Susan Novotny, Gillette Children's Specialty Healthcare, USA

Panagiotis Ntziachristos, Northwestern University, USA

Stefan Oehlers, Centenary Institute, Australia

Naoki Okamoto, University of California, Riverside, USA

Peter Olinga, University of Groningen, The Netherlands

Heymut Omran, University Hospital Münster, Germany

Alvaro Ordonez, Johns Hopkins University, USA

Carolina Ortiz Cordero, University of Minnesota, USA

Daniel Ory, Washington University School of Medicine, USA

Michael Pack, Uinversity of Pennsylvania, USA

Antonio Pagán, Cambridge University, UK

Eirini Papapetrou, Icahn School of Medicine at Mount Sinai, USA

Carmen Paradas, Hospital Universitario Virgen del Rocío, Spain

Raghuveer Parthasarathy, University of Oregon, USA

Jose Pastor-Pareja, Tsinghua University, China

Ketan Patel, University of Reading, UK

Graham Pavitt, University of Manchester, UK

Per Pedersen, University of Copenhagen, Denmark

Rita Perlingeiro, University of Minnesota, USA

Christine Petit, Institut Pasteur & College de France, France

Michale Petris, University of Missouri, USA

Tatiana Petrova, Ludwig Institute for Cancer Research, Switzerland

Dana Philpott, The Hospital for Sick Children, Canada

Stefano Piccolo, University of Padua, Italy

Marita Pietrucha-Dutczak, Medical University of Silesia, Poland

Tatjana Piotrowski, Stowers Institute, USA

Steve Pollard, University of Edinburgh, UK

Cristina Porcheri, University of Zurich, Switzerland

Rebecca Poulos, University of Sydney, Australia

Xavier Prieur, University of Nantes, France

Sonja Pyott, University of Groningen, The Netherlands

Nidia Quillinan, University of Colorado, Anschutz Medical Campus, USA

Anjana Ramdas Nair, NYU Abu Dhabi, Abu Dhabi

Tennore Ramesh, University of Sheffield, UK

John Rawls, University of North Carolina School of Medicine, USA

Adriana Rebelo, University of Miami, USA

Francesco Retta, University of Torino, Italy

Hamidreza Riazifar, UCI, USA

Saima Riazuddin, University of Maryland, USA

Carlo Rinaldi, University of Oxford, UK

Lee Roberts, University of Leeds, UK

Craig Robson, Newcastle University, UK

Jason Rosch, St. Jude Children's Research Hospital, USA

Alan Rosenberg, University of Saskatchewan, Canada

Emily Rosowski, Clemson University, USA

Filippo Rosselli, CNRS Institut Gustave Roussy, France

Camilo Ruiz-Bedoya, Johns Hopkins University, USA

Paola Rusmini, The Centre of Excellence for Neurodegenerative Diseases, University of Milan, Italy

Hyung Don Ryoo, New York University School of Medicine, USA

Kirsten Sadler Edepli, New York University Abu Dhabi, United Arab Emirates

Alvaro Sagasti, University of California, Los Angeles, USA

Sourav Saha, NIH, USA

Erik Sahai, Francis Crick Institute, UK

Yukio Saijoh, University of Utah, USA

Jean-Pierre Saint-Jeannet, New York University, USA

Valerie Sampson, Alfred I. du Pont Hospital for Children, USA

Berta Sanchez-Laorden, Spanish Research Council, Spain

Pamela Santonicola, IBBR CNR, Italy

Emiko Sato, Tohoku University, Japan

Miriam Schmidts, University of Freiburg Medical Center, Germany

Benedikt Schoser, Friedrich-Baur-Institut, Germany

Oren Schuldiner, Weizmann Institute of Science, Israel

Stefan Schulte-Merker, Hubrecht Institute (KNAW), The Netherlands

Daryl Scott, Baylor College of Medicine, USA

Julie Secombe, Albert Einstein College of Medicine, USA

Cheryle Seguin, The University of Western Ontario, Canada

Praveen Sethupathy, Cornell University, USA

Le Shen, University of Chicago, USA

Zhongfang Shi, Capital Medical University, China

Celia Shiau, University of North Carolina at Chapel Hill, USA

Donghun Shin, University of Pittsburgh, USA

Cheryl Shoubridge, University of Adelaide, Australia

Joshua Shulman, Baylor College of Medicine, USA

Eric Shusta, University of Wisconsin, USA

Ody Sibon, University of Groningen, The Netherlands

Florian Siebzenrubl, Cardiff University School of Biosciences, UK

Dirk Sieger, University of Edinburgh, UK

Detlef Siemen, University of Magdeburg, Germany

Dhiraj K. Singh, Cornel University, USA

Karim Si-Tayeb, University of Nantes, France

John Sled, Hospital for Sick Children, Canada

Ian Smyth, Monash University, Australia

Charlotte Sørensen, Aarhus University, Denmark

Pietro Spitali, Leiden University Medical Center, The Netherlands

Ferdinando Squitieri, Mendel Institute of Human Genetics, Italy

Jemeen Sreedharan, King's College London, UK

David Stanek, Institute for Molecular Genetics of the Czech Academy of Sciences, Prague, Czech Republic

Thaddeus Stappenbeck, Washington University School of Medicine, USA

Michelle Starz-Gaiano, University of Maryland, USA

Richard Steet, Greenwood Genetic Center, USA

Werner Stenzel, Charité, Berlin, Germany

Rodney Stewart, University of Utah, USA

Cheryl Stoddart, University of California, San Francisco, USA

Helen Stolp, Royal Veterinary College, UK

Erik Storkebaum, Radboud University, The Netherlands

Lisa Stubbs, University of Illinois, USA

Jung-Joon Sung, Seoul National University Hospital, Republic of Korea

Neil Surana, Duke University School of Medicine, USA

Piyumika Suriyampola, Arizona State University, USA

Masatoshi Suzuki, University of Wisconsin, USA

Amanda Swain, Institute of Cancer Research, UK

Takanori Takebe, Cincinnati Childrens' Hospital, USA

Yoichiro Tamori, Hokkaido University, Japan

Bertrand Tavitian, Paris Descartes University, France

Rohan Teasdale, University of Queensland, Australia

Avinash Thakur, University of British Columbia, Canada

Michael Themis, Brunel University, UK

Jennifer Thies, The University of Alabama, USA

Glen Tibbits, Simon Fraser University, Canada

Vincent Timmerman, University of Antwerp, The Netherlands

Natascia Tiso, University of Padova, Italy

David Tobin, Duke University Medical Center, USA

Alessio Torcinaro, Sapienza Universita di Rome, Italy

Paul Trainor, Stowers Institute for Medical Research, USA

Eirini Trompouki, Max Planck Institute of Immunology and Epigentics, Germany

Maja Trost, University Medical Centre Ljubljana, UK

Leo Tsuda, National Institute for Longevity Sciences, Japan

Valter Tucci, Istituto Italiano di Tecnologia, Italy

Abigail Tucker, King's College London, UK

Anthony Turner, University of Leeds, UK

Pravesh Tyagi, University of Canterbury, New Zealand

Gokhan Unlu, Rockfeller University, USA

Cyrille Vaillend, University Paris-Sud, France

Robert van de Ven, University Medical Center Utrecht, The Netherlands

Fredericus van Eeden, The University of Sheffield, UK

Tjakko van Ham, Erasmus MC, The Netherlands

Pieter Van Vlierberghe, Ghent University, Belgium

Peter Vangheluwe, KU Leuven, Belgium

Brian Varisco, Cincinnati Children's Hosptial, USA

Neeti Vashi, Hospital for Sick Children, Canada

Anju Vasudevan, Harvard Medical School, USA

Alexej Verkhratsky, University of Manchester, UK

Julien Vermot, IGBMC, France

Cindy Voisine, Northeastern Illinois University, USA

Henry Waldvogel, University of Auckland, New Zealand

Lucas Waltzer, Universite Clermont Auvergne, CNRS, France

Jian Wang, Dalhousie University, Canada

Jiaxing Wang, Emory University, USA

Xu Wang, Metabolism and Molecular Medicine, Ministry of Education, China

Michael Wangler, Baylor College of Medicine, USA

Jennifer Watts, Washington State University, USA

Hans Weiher, Hochschule Bonn-Rhein-Sieg, Germany

Chris Weihl, Washington University School of Medicine, USA

Dominic Wells, Royal Veterinary College, UK

Wolfgang Weninger, Medical University of Vienna, Austria

Robert Wessells, Wayne State University, USA

Robert Wheeler, University of Maine, USA

Tanya Whitfield, University of Sheffield, UK

Rebecca Wingert, University of Notre Dame, USA

Valerie Wittamer, Université Libre de Bruxelles, Belgium

Bogdan Wlodarczyk, Baylor College of Medicine, USA

Adam Wong, University of Florida, USA

Ian Wood, University of Leeds, UK

Anping Xia, Stanford University, USA

Lei Xue, Tongji University, China

Shu Yang, NIH, USA

Pamela Yelick, Tufts University, USA

Mervin Yoder, Indiana University, USA

Mark Yorek, University of Iowa, USA

H. Joseph Yost, University of Utah, USA

Min Yu, University of Southern California, USA

Catherine Yzydorczyk, University of Lausanne, Switzerland

Eldad Zacksenhaus, University of Toronto, Canada

Stephane Zaffran, Aix-Marseille University, France

Daniela Zarnescu, University of Arizona, USA

Frank Zaucke, University of Koln, Germany

Sheng Zhang, University of Texas, USA

Yong Zhang, Chinese Academy of Sciences, China

Wei-jiang Zhao, Shantou University Medical College, China

Wei Zheng, National Center for Advancing Translational Sciences (NCATS), NIH, USA

Gong Zhiyuan, National University of Singapore, Singapore

Chengji Zhou, University of California at Davis, USA

Weibin Zhou, Icahn School of Medicine at Mount Sinai, USA

Yang Zhou, Brown University, USA

Berislav Zlokovic, University of Southern California, USA

Gulab Zode, University of North Texas Health Science Center, USA
